# Orphan Three-Finger Toxins Bind at Tissue Factor–Factor VIIa Interface to Inhibit Factor X Activation: Identification of Functional Site by Docking

**DOI:** 10.1055/s-0038-1672184

**Published:** 2018-09-26

**Authors:** Manisha Choudhury, Ryan J. R. McCleary, R. Manjunatha Kini, Devadasan Velmurugan

**Affiliations:** 1 CAS in Crystallography and Biophysics, University of Madras, Chennai, Tamil Nadu, India; 2 Department of Biological Sciences, National University of Singapore, Singapore, Singapore; 3 Department of Biology, Stetson University, DeLand, Florida, United States

**Keywords:** extrinsic tenase complex, anticoagulant, factor VIIa–tissue factor complex inhibitor, orphan group I toxins

## Abstract

Three-finger toxins (3FTxs) contribute to toxicity of venomous snakes belonging to the family Elapidae. Currently, functions of a considerable proportion of 3FTxs are still unknown. Here, we describe the function of orphan group I 3FTxs consisting of four members. We also identified a new member of this group by sequencing a transcript isolated from *Naja naja* venom. This transcript, named najalexin, is identical to that previously described 3FTx from *Naja atra* venom gland, and shared high sequence identity with ringhalexin from *Hemachatus haemachatus* and a hypothetical protein from *Ophiophagus hannah* (here named as ophiolexin). The three-dimensional structure, as predicted by molecular modeling, showed that najalexin and ophiolexin share the same conserved structural organization as ringhalexin and other 3FTxs. Since ringhalexin inhibits the activation of factor X by the tissue factor–factor VIIa complex (TF-FVIIa), we evaluated the interaction of this group of 3FTxs with all components using *in silico* protein–protein docking studies. The binding of orphan group I 3FTxs to TF-FVIIa complex appears to be driven by their interaction with TF. They bind to fibronectin domain closer to the 170-loop of the FVIIa heavy chain to inhibit factor X activation. The docking studies reveal that functional site residues Tyr7, Lys9, Glu12, Lys26, Arg34, Leu35, Arg40, Val55, Asp56, Cys57, Cys58, and Arg65 on these 3FTxs are crucial for interaction. *In silico* replacement of these residues by Ala resulted in significant effects in the binding energies. Furthermore, these functional residues are not found in other groups of 3FTxs, which exhibit distinct pharmacological properties.

## Introduction

Snake venom is an adaptive evolutionary innovation that consists of a mixture of proteins and polypeptides, a large number of which exhibit diverse biological activities. This venom gland secretion usually contains proteins that belong to various structural protein superfamilies such as the three-finger toxins (3FTxs), phospholipases A _2_ , C-type lectin-like proteins, serine proteases, and metalloproteinases. 1 2 3 The diversity of snake venom toxins is an outcome of evolutionary processes, which involve duplication of toxin-encoding genes followed by structural and functional diversification. 4 5 6 7 The latter diversification steps are thought to be due to faster rates of sequence evolution. 8 9 10 11 Thus, the multiplicity of toxins with diverse actions encoded by multigene families is a common theme in venom evolution.

3FTxs are a family of nonenzymatic polypeptides generally composed of 60–74 amino acid residues, and they have been widely studied. 1 12 13 Their presence has been reported in elapid (both terrestrial [elapine] and aquatic [hydrophiine]) subfamilies) and colubrid venoms 14 15 as well as in viperid venoms. 16 3FTxs contain four to five disulfide bridges, of which four are conserved. Thus, there is a typical pattern of protein folding in this family of toxins, whereby three β-stranded loops extend from a central core that contains the four conserved disulfide bridges, and resemble a hand with three protruding fingers. 1 12 17 18 Despite this similarity in structure, 3FTxs have a wide range of functional diversity. 1 12 Based on their function, 3FTxs can be broadly categorized as neurotoxins, 19 cardiotoxins/cytotoxins, 20 acetylcholinesterase inhibitors, 21 L-type calcium channel blockers, 22 platelet aggregation inhibitors, 23 anticoagulants, 24 and β-cardiotoxins. 25 Thus, 3FTxs have been extensively used as investigational ligands, resulting in characterization of a large number of them.

We performed a phylogenetic analysis of 3FTxs and showed a much greater diversity of family members than was previously known. 26 A considerable proportion of 3FTxs belonged to clades with unknown function. Accordingly, we identified 20 orphan groups containing 67 individual toxins. 26 These orphan groups were defined through comparisons of consensus sequences, physical properties, and detection of known functional motifs. The functional analyses of these orphan groups are crucial, as these groups may contain novel toxins that have interesting pharmacological properties and distinct protein targets thereby allowing their use as investigational tools. Previously, we have characterized the structural and functional properties of some of these orphan 3FTxs, including ringhalexin (orphan group I), 27 candoxin (orphan group IV), 28 29 30 bucandin (orphan group XIX), 31 32 and exactin (orphan group XX). 33

When it was first described in 2003, the orphan group I 3FTxs comprised a single member identified as neurotoxin-like protein from *Naja atra* venom (NTL2) (NCBI accession Q9W717). 26 During the genomic study of the king cobra ( *Ophiophagus hannah* ) from Indonesia, we identified a second 3FTx transcript that encoded a protein that showed 84% identity to NTL2 (NCBI accession ETE58964.1). 34 An identical but partial transcript was also identified in the venom glands of a king cobra from Malaysia. 35 Recently, we characterized the structure and function of ringhalexin, a protein from *Hemachatus haemachatus* venom (NCBI accession C0HJT5.1), 27 that exhibits 94% identity (98% similarity) with NTL2. Despite its highly similar three-dimensional structure compared with other classes of 3FTxs, ringhalexin shows potent anticoagulant activity and inhibits the extrinsic tenase complex comprising tissue factor–factor VIIa (TF-FVIIa) that is involved in the activation of factor X to factor Xa. Affinity of ringhalexin toward the catalytic TF-FVIIa is two times higher than toward the enzyme–substrate complex TF-FVIIa-FX (84.25 ± 3.53 nM compared with 152.5 ± 11.32 nM). 27

Extensive studies have been conducted on the structure–function relationships of the TF-FVIIa complex. Site-directed mutagenesis for identification of the binding site on TF for FVIIa revealed that six discontinuous regions of TF (residues 16–20, 40–46, 60–69, 101–111, 129–151, 193–207) were crucial for interaction with FVIIa. 36 These residues were classified into two groups. The first group (residues Lys46, Gln110, Arg135, Phe140, Val207) was found to be important only for interactions with FVIIa, and the second group (residues Lys20, Asp44, Trp45) were required to induce the conformational change in FVIIa for enhanced activity. 36 Furthermore, it was reported that the discontinuous binding site for FVIIa is located at the domain–domain interface and also includes residues from extended loops and β-strands. 37 Alanine-scanning mutagenesis of the binding site residues on TF, including the residues within the flanking β-strands, showed that three residues within strand C (Tyr34, Gln37, Ile38) and two residues within C' (Lys48, Tyr51) were important for TF cofactor function. 37 Furthermore, site-directed Ala exchanges in the strand-loop-strand structure showed that TF residues 157–167 are crucial for functional interactions. 38 Earlier reports also showed that the area for interaction of TF with FVIIa extends from the cleft formed by the two structural modules including residues Lys20, Ile22, Lys48, Asp58, Arg135, and Phe140 to the edge of the three- and four-stranded sheets composed of hydrophobic side chains in the amino-terminal module (residues Gln37, Asp44, Trp45, Phe76, Tyr78). 39 The first epidermal growth factor domain of FVIIa (residues Gln64, Ile69, Phe71, and Arg79) and the protease domain (Arg277, Met306, Asp309) form energetically important binding contacts located at the interface with TF. 40 The Phe225 residue position plays a crucial role in the allosteric network. 41

During our proteomic characterization of *N. naja* venom, 42 we identified three peptides that perfectly match segments from NTL2 (27KFPK30, 41GCAATCPKAEAR52, and 53VYVDCCAR60). Here, we describe the complete sequence of a 3FTx transcript isolated from *N. naja* venom that is 100% identical to NTL2. Based on high identity and similarity among the four proteins from the *N. naja* , *N. atra* , *O. hannah* , and *H. haemachatus* venoms mentioned earlier, we hypothesized that they would belong to 3FTx orphan group I and exhibit similar three-dimensional structures and anticoagulant activities as ringhalexin. Therefore, we named these 3FTxs from *N. atra* , *N. naja* , and *O. hannah* venoms as natralexin, najalexin, and ophiolexin, respectively.

To understand the interaction of ringhalexin and other orphan group I 3FTxs with coagulation factors involved in the extrinsic tenase complex and to determine structure–function relationships of these toxins, we used molecular docking experiments. Likewise, to this end, we modeled the structures of najalexin and ophiolexin based on the crystal structure of ringhalexin (PDB code 4ZQY) 27 and studied the interaction of these proteins with FVIIa, TF, FX, and TF-FVIIa using *in silico* protein–protein docking approaches. These studies showed that these toxins bind at the interface of TF-FVIIa and inhibit FX activation. Furthermore, *in silico* mutation of amino acid residues was performed to understand the effect on the binding affinity of the native and mutated complex. The specificity of these toxins toward TF-FVIIa appears to be contributed by residues Tyr7, Lys9, Glu12, Lys26, Arg34, Leu35, Arg40, Val55, Asp56, Cys57, Cys58, and Arg65 that are not found in other 3FTxs.

## Materials and Methods

### Venoms

Lyophilized crude *N. naja* venom (pooled) was purchased from the Irula Snake Catchers' Society, Tamil Nadu, India. Three to four snakes were caught from the forests within Tamil Nadu and held in captivity for 2 to 6 weeks. During this period, venom was extracted from the snakes about two to four times.

### Total RNA Extraction from Crude Venom and cDNA Synthesis

RNA was extracted from 2 mg of lyophilized venom utilizing 1.0-mL TRIzol. After incubation for 5 minutes at room temperature (∼25°C), 200-µL chloroform was added, and the sample was centrifuged at 12,000 g for 15 minutes. The aqueous upper phase was transferred to a new RNAse-free microcentrifuge tube, and 400 µL of 100% isopropanol were added to precipitate RNA. The tube was incubated for 10 minutes at room temperature and centrifuged at 12,000 g for 10 minutes. The supernatant was discarded and the resulting RNA pellet washed with 800 µL of 70% ethanol. Total RNA concentrations were determined using a NanoVue (GE Healthcare, Uppsala, Sweden) and a Qubit 2.0 Fluorometer (Life Technologies, Camarillo, California, United States). cDNA synthesis from total RNA was performed using the ExactSTART Eukaryotic mRNA 5′- and 3′-RACE (Rapid Amplification of cDNA Ends) Kit (Epicentre, Madison, Wisconsin, United States) following the manufacturer's protocols. Reverse transcriptase cDNA synthesis was initiated by the oligo (dT) adaptor primer provided with the kit and effectively selected for polyadenylated mRNAs.

### 5′- and 3′-RACE

The conserved 3FTx signal peptide sequence was used to design the sense primer sequence (5′-AGATGAAAACTCTGCTGCTGTCCTTGGT-3′), and the universal antisense primer provided with the kit was also used. Polymerase chain reaction (PCR) high-fidelity polymerase (3 μL, KAPA HiFi) was used with 1 to 2 µL of cDNA template and 1 µL of both forward and reverse primers. Touchdown amplification was performed as follows: initial denaturation at 95°C for 3 minutes; 40 cycles of 98°C for 20 seconds, 54°C for 45 seconds, and 72°C for 1 minute; and a final extension at 72°C for 10 minute. Amplicons were visualized on a 1% TAE/agarose gel. Furthermore, we performed PCR amplification of the neurotoxin-like protein transcript using gene-specific sense (5′-CAGGTTATGTCTCTCAGACTACTCAATAT-3′) and antisense (5′-CACAACAATCAACATACACACGGGCTT-3′) primer sequences.

### Cloning and Sequencing of Venom cDNA

The amplified and purified najalexincDNA from *N. naja* venom was ligated into the pSK+ vector and further subcloned in the pQE30 vector at *Bam* HI and *Hind* III sites. The forward and reverse primers used were 5′-GATCCATGAAAACTCTGC-3′ and 5′-AGCTTCTATCGGTTGC-3′, respectively. The resulting constructs were transfected into *Escherichia coli* DH5α competent cells following the manufacturer's protocol. Transformed *E. coli* were grown on nutrient-rich agar plates overnight at 37°C with ampicillin (100 µg/L), isopropyl beta-D-1-thiogalactopyranoside (0.1 M), and X-gal (20 mg/mL) for white/blue colony selection. Selection of recombinant plasmids from agar plates resulted in 30 *E. coli* clones. Each recombinant colony was placed in 2-mL lysogeny broth with 1 µL/mL ampicillin and shaken overnight at 180 rpm and 37°C. The Quick Clean 5M Miniprep Kit (GenScript, Piscataway, New Jersey, United States) was used for purifying plasmid copies for each *E. coli* colony, and further sequencing was performed on an Applied Biosystems 3500 Sequence Analysis instrument.

### Sequence Alignment and Phylogenetic Analysis

The full-length coding sequence was submitted to NCBI (nucleotide accession: KX657840; protein accession: APB88857). 3FTx homologs that showed high homology from a BLAST search were used for phylogenetic analysis and tree building. The multiple sequence alignment was performed and visualized using ESPript 43 and the phylogenetic tree was constructed using MEGA v6.06. 44

### Homology Modeling and Validation

Homology models for najalexin and ophiolexin were built using Prime 3.1 in Schrödinger Suite (Schrödinger, LLC, New York, NY, United States). In this approach, najalexin (94% sequence identity to ringhalexin) and ophiolexin (83% sequence identity to ringhalexin) sequences were used as targets, and ringhalexin from *H. haemachatus* (PDB code 4ZQY) was used as template for building the model. The validations of the models were performed using the Phi–Psi stereochemical profile of the Ramachandran plot from the PROCHECK program in the SAVES metaserver. 45

### Molecular Protein–Protein Docking

As mentioned, ringhalexin is a mixed-type inhibitor of the extrinsic tenase complex. 27 It binds to catalytic TF-FVIIa better than TF-FVIIa-FX enzyme–substrate complex, but does not inhibit FVIIa or FX alone. 27 To understand the molecular recognition and selectivity, we used *in silico* protein–protein docking of ringhalexin and two closely related proteins. We performed docking studies with human FVIIa, TF, TF-FVIIa (all from PDB code 2ZWL), and FX (PDB code 5K0H). FVIIa, TF, TF-FVIIa, FX, najalexin, ringhalexin, and ophiolexin models were constructed using the protein preparation wizard (Schrödinger) for adding hydrogen and removing steric hindrances. Rigid protein–protein docking was performed using the online ClusPro program. 46 The top-ranked binding poses were used for calculating binding free energy using the approach utilizing molecular mechanics, the generalized Bom model, and solvent accessibility in the Embrace minimization module (Schrödinger). A Polak–Ribière conjugate gradient 47 energy optimization method with an OPLS2005 force field was used for calculation of gas phase energy and the generalized Born and surface area continuum solvation method was used for calculation of the solvation phase energy for each component of the molecular complex:

Δ *E* = *E*_complex_ – *E*_ligand_ – *E*_protein_

The docked poses for ringhalexin, najalexin, and ophiolexin with FVIIa, TF, TF-FVIIa, and FX having the most favorable binding free energies were considered for H-bond and salt bridge interaction analyses.

### Analyses of Mutational Effects on the Complexes

Residues involved in interactions of each ringhalexin, najalexin, and ophiolexin with TF and TF-FVIIa were mutated to Ala or Gly to analyze the differences in affinity of the native and mutant protein to the complex. The interacting Cys residues were not mutated as they were involved in the disulfide bridge formations. The residue scanning module of BioLuminate in Schrödinger was used for mutation analysis of the complex (Biologics Suite 2014–1: BioLuminate, Schrödinger) in terms of Δ binding affinity which represents differences in binding free energy between the native and mutated protein complex.

## Results and Discussion

### Sequence of Najalexin

**Fig. 1:**
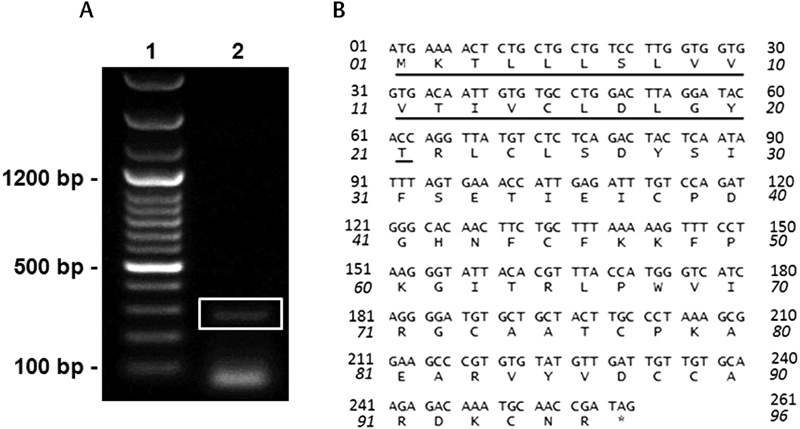
NajalexincDNA transcripts amplified from *Naja naja* venom. ( **A** ) Agarose gel electrophoresis showing najalexincDNA transcripts amplified from venom-derived mRNA in *Naja naja* venom (Lane 1: DNA ladder, lane 2: amplified najalexin transcript [boxed]). ( **B** ) cDNA and deduced amino acid sequence of najalexin. The signal peptide consisting of 21 amino acid residues is underlined. The asterisk denotes the stop codon.

**Fig. 2:**
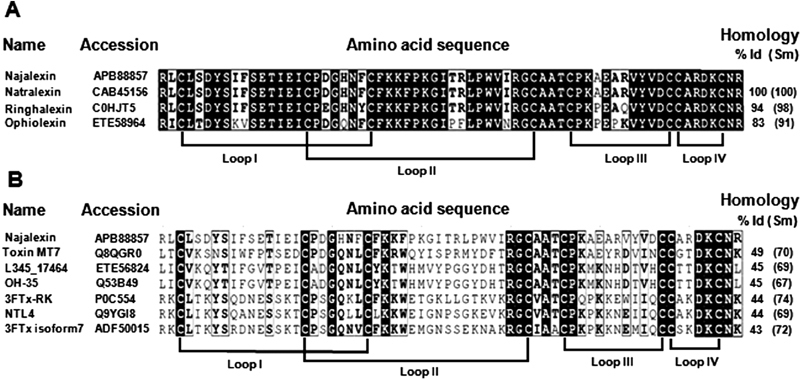
Multiple sequence alignment of najalexin with 3FTxs. ( **A** ) Identity and similarity among orphan group I 3FTxs. ( **B** ) Similarity with other closely related 3FTx homologues obtained from BLAST search against the NCBI database. Toxin names, accession numbers, and amino acid sequence along with percentage of identity and similarity of each protein sequence compared with najalexin are shown. Conserved residues in all sequences are highlighted in black and the disulfide bridges and loops are marked.

We obtained 30 positive clones of najalexin from *N. naja* venom ( Fig. 1A ) and determined their full-length nucleotide sequences. This full-length sequence was aligned with natralexin ( Supplementary Fig. S1 ). The open reading frame is 261 bp in length, encodes for a precursor protein with a 21-residue signal peptide, and yields a mature protein of 65 amino acid residues (average mass: 7,409.68 Da) ( Fig. 1B ). The complete cDNA and deduced protein sequences of najalexin were submitted to the NCBI database (accession numbers: KX657840 and APB88857, respectively). The mature protein sequences of najalexin and natralexin are 100% identical to each other and showed 94% identity to ringhalexin isolated from *H. haemachatus* venom and 83% identity to ophiolexin ( Fig. 2 ). It was observed that the residues responsible for recognizing the nicotinic acetylcholine receptor (His6, Gln7, Ser9, Tyr25, Trp29, Lys27, Asp31, Phe32, Gly34, Ile36, and Glu38) 48 49 were missing in these 3FTxs. Thus, neurotoxin-like protein is a misnomer for this group of 3FTxs.

The BLAST and phylogenetic analysis ( Supplementary Fig. S2 ) revealed that najalexin, ringhalexin, and ophiolexin form a cluster (orphan group I). Other related toxins including MT7 (NCBI: Q8QGR0) and L345_17464 (NCBI: ETE56824) show less than 50% identity ( Fig. 2 ).

### Modeled Structures of Najalexin and Ophiolexin

**Fig. 3:**
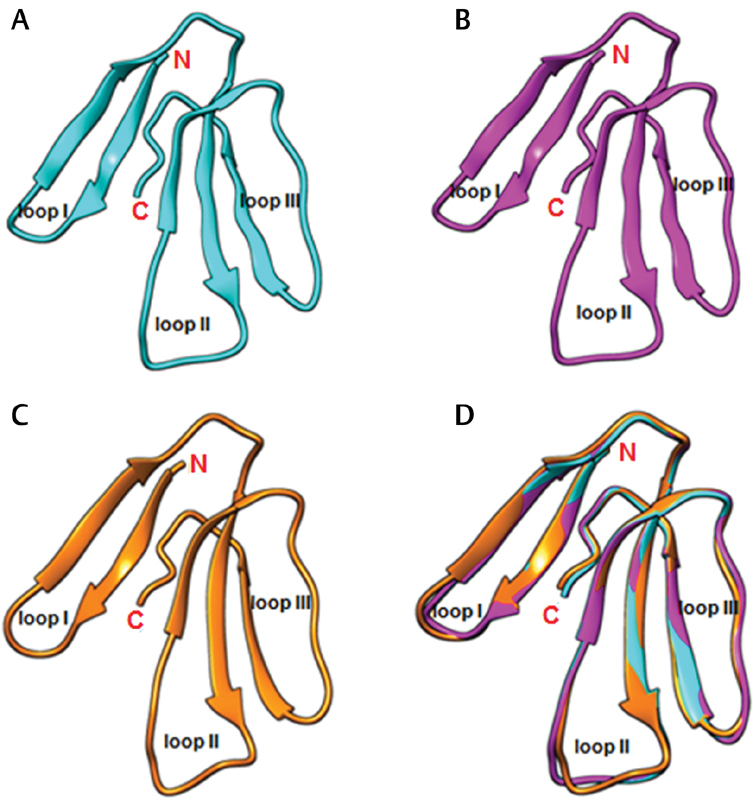
Three-dimensional structures of toxins. ( **A** ) Crystal structure of ringhalexin (PDB: 4ZQY). Three-dimensional models of ( **B** ) najalexin and ( **C** ) ophiolexin were constructed using ringhalexin structure as the template. ( **D** ) Superimposed structures of ringhalexin ( *cyan* ), najalexin ( *magenta* ), and ophiolexin ( *orange* ). The loops and N- and C-terminals are labeled. Root mean square deviation (RMSD) between najalexin and ringhalexin is 0.035 Å, between najalexin and ophiolexin is 0.771 Å, and between ringhalexin and ophiolexin is 0.769 Å.

The three-dimensional model structures of najalexin and ophiolexin were constructed using the crystal structure of ringhalexin (PDB code 4ZQY) as the template. The individual and superimposed structures of ringhalexin, najalexin, and ophiolexin are shown in Fig. 3 . The stereochemical parameters of these models were analyzed by PROCHECK and all residues are in the allowed regions of the Ramachandran plot ( Supplementary Fig. S3 ). The Ramachandran map statistics revealed that 80 and 20% of the residues lie in the most favored and additionally allowed regions for ringhalexin, respectively, while these statistics are 89.3 and 10.7% in najalexin and 86.8 and 13.2% in ophiolexin. The root mean square deviation (RMSD) for heavy (main chain) atoms between ringhalexin and najalexin is 0.035 Å, between najalexin and ophiolexin is 0.771 Å, and between ringhalexin and ophiolexin is 0.769 Å.

### Protein–Protein Docking

Recently, we described the function of ringhalexin, an anticoagulant that inhibits FX activation through the extrinsic tenase complex made up of TF, FVIIa, and Ca ^2+^ ions. 27 Ringhalexin shows two times higher affinity toward TF-FVIIa than toward the TF-FVIIa-FX complex. However, the molecular details of interaction with coagulation factors in the TF-FVIIa and the structure–function relationship of ringhalexin and other related toxins were not clear.

#### TF Drives the Binding to Extrinsic Tenase Complex

**Fig. 4:**
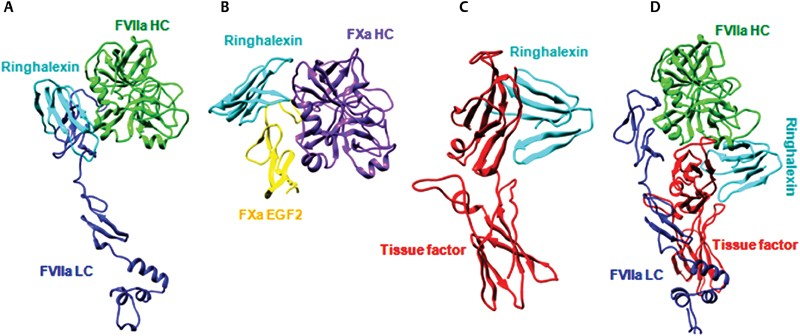
Molecular docking of ringhalexin with coagulation factors of the extrinsic tenase complex. Interactions of ringhalexin with ( **A** ) FVIIa, ( **B** ) FX, ( **C** ) TF, and ( **D** ) TF-FVIIa. HC, heavy chain; LC, light chain.

The toxins docked with FVIIa and FX showed interactions at the interface of the heavy (H) and light (L) chains but on the opposite surface of their active sites ( Fig. 4A , B ; see also Supplementary Fig. S4A , B , E , F ; Table 1 ). Thus, this binding may not affect the activity of FVIIa or FX, as was experimentally observed. 27 Furthermore, the Δ total binding free energies of ringhalexin, najalexin, and ophiolexin docked with TF ( Table 2 ) were comparatively higher than that of toxins docked with TF-FVIIa complex ( Table 3 ).

**Table 1:** Δ total binding free energy (kcal/mol) for the ringhalexin, najalexin, and ophiolexin docked with FVIIa and FX

Toxin	ΔTotal binding free energy (kcal/mol)	
	FVIIa	FX
Ringhalexin	−155.415	−168.315
Najalexin	−206.399	−221.112
Ophiolexin	−177.837	−204.445

Note: The first column indicates the orphan group I toxins. The second column lists the Δ total binding free energies on docking each toxin with FVIIa (heavy chain) (PDB code 2ZWL). The third column lists the Δ total binding free energies on docking each toxin with FX (PDB code 5K0H).

**Table 2:** Residues of ringhalexin, najalexin, ophiolexin, and TF (tissue factor) involved in interactions based on protein–protein docking studies

Complex	Loop I	Loop II	Loop III	Δtotal binding free energy (kcal/mol)	Buried (%)
Ringhalexin–TF		Lys26–Glu95 (SB) **Lys30–Glu91 (SB)** Arg34–Thr40 (SB)	**Cys63–Asn11 (HB)** Arg65–Asn11 (HB)	−76.612	47.53
Najalexin–TF		Lys26–Glu95 (SB) **Lys30–Glu91 (SB)** Arg34–Thr40 (SB)	Tyr54–Pro92 (HB) Arg60–Tyr10 (HB) **Cys63–Asn11 (HB)** Arg65–Thr13 (HB)	−82.188	50.51
Ophiolexin–TF	Tyr7 **–** Thr13 (HB) Lys9–Glu99 (SB)	Lys26–Ala9 (SB) **Lys30–Glu91(SB)** Pro33–Arg74 (SB)	Tyr54–Tyr94 (HB) **Cys63–Asn11 (HB)**	−94.731	51.56

Abbreviations: HB, hydrogen bond; SB, salt bridge.

Note: The first column indicates the complex between orphan group I toxins and TF. The second, third, and fourth columns list the residues of loop I, loop II, and loop III of toxins, respectively, involved in H-bond or salt bridge interactions with TF residues. The fifth column lists the Δ total binding free energies on docking each toxin with TF (PDB code 2ZWL). The sixth column lists the percentage of buried residues of toxin in each complex.

**Table 3:** Residues of ringhalexin, najalexin, ophiolexin, and TF-FVIIa involved in interactions based on protein–protein docking studies

Complex	Loop I	Loop II	Loop III	Δtotal binding free energy (kcal/mol)	Buried (%)
Ringhalexin–TF-FVIIa	Tyr7–Ser97 (T) (HB) Ile9–Glu99 (T) (HB)	Tyr23–Arg170C (H) (HB) Arg34–Asp66 (T) (HB) Leu35–Lys68 (T) (HB) Arg40–Glu99 (T) (HB)	Tyr54–Tyr184 (H) (HB) Val55–Ser185 (H) (HB) Asp56–Ser185 (H) (HB) Arg65–Gln170 (H) (HB)	−211.326	49.29
Najalexin–TF-FVIIa	Tyr7–Glu99 (T) (HB)	Lys26–Glu99 (T) (HB) Arg34–Asp66 (T) (HB) Leu35–Lys68 (T) (HB) Arg40–Glu99 (T) (HB)	Val55–Ser185 (H) (HB) Asp56–Ser185 (H) (HB) Cys57–Ser185 (H) (HB) Cys58–Gln170 (H) (SB) Ala59–Gln170 (H) (HB) Arg65–Glu95 (T) (HB)	−305.777	51.78
Ophiolexin–TF-FVIIa	Tyr7–Glu99 (T) (HB) Lys9–Trp14 (T) (HB) Glu12–Asn11(T) (HB)	Lys26–Glu99 (T) (HB)	Lys48–Ser188A (H) (HB) Cys57–Ser185 (H) (HB) Cys58–Gln170 (H) (SB) Ala59–Gln170 (H) (HB) Lys62–Glu95 (T) (HB) Arg65–Gln170 (H) (HB)	−379.706	53.18

Abbreviations: H, FVIIa heavy chain; HB, hydrogen bond; SB, salt bridge; T, tissue factor.

Note: The first column indicates the complex between orphan group I toxins and TF-FVIIa. The second, third, and fourth columns list the residues of loop I, loop II, and loop III of toxins, respectively, involved in H-bond or salt bridge interactions with TF-FVIIa residues. The fifth column lists the Δ total binding free energies on docking each toxin with TF-FVIIa (PDB code 2ZWL). The sixth column lists the percentage of buried residues of toxin in each complex.

It was observed from the docking results of ringhalexin with TF and TF-FVIIa that the interaction surface with TF remains the same, but its interaction surface with FVIIa changed drastically to the opposite surface ( Fig. 4C , D compared with Fig. 4A ). Since the orientation of ringhalexin docked with TF and TF-FVIIa remains the same but with a distinct change in the binding surface of FVIIa, we concluded that TF drives the interaction of ringhalexin to TF-FVIIa. Similarly, on docking each najalexin and ophiolexin to TF and TF-FVIIa, it was observed that the binding site was similar to ringhalexin ( Supplementary Fig. S4C , D , G , H ). The orientation of the toxins with TF and TF-FVIIa remains the same but distinct change is observed in the binding surface of FVIIa. We, therefore, concluded that TF drives the interaction of these toxins with TF-FVIIa complex.

#### Orientation of the Toxins in Complex with TF and TF-FVIIa

**Fig. 5:**
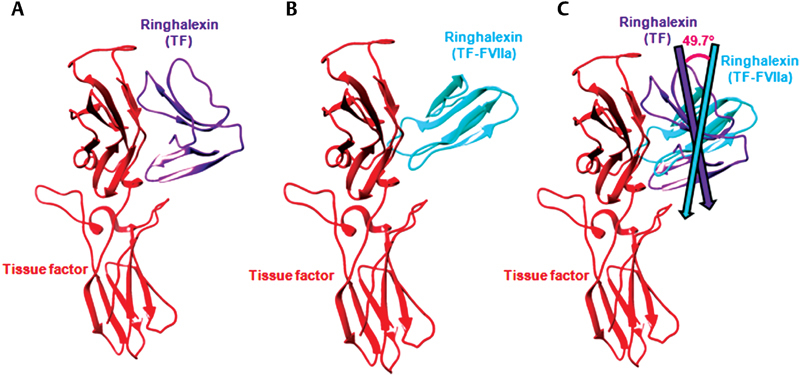
Change of orientation between ringhalexin docked with TF and TF-FVIIa. Binding poses of ringhalexin to ( **A** ) TF and ( **B** ) TF-FVIIa. ( **C** ) Superimposition of binding poses of ringhalexin docked with TF and TF-FVIIa results in an RMSD 0.727 Å. The vectors passing through bound ringhalexin to TF and TF-FVIIa make an angle of 49.7°.

The binding poses of ringhalexin docked with TF and TF-FVIIa indicate change in orientation of the toxin in these complexes. Superimposition of ringhalexin in these two complexes showed an RMSD of 0.727 Å, and the toxin molecules deviated from each other by 49.7° ( Fig. 5 ). This change in orientation of the toxin on binding to TF-FVIIa compared with only TF is due to the “molecular push” implied on the toxin by FVIIa heavy chain. Similarly, superimposing the binding poses of najalexin docked with TF and TF-FVIIa resulted in an RMSD of 0.714 Å, and 59.4° deviation ( Supplementary Fig. S5A — C ). Ophiolexin showed an RMSD of 0.742 Å and 52.7° deviation when the docked poses with TF and TF-FVIIa were superimposed ( Supplementary Fig. S5D — F ).

The orientation differences between the binding poses of the three toxins docked with TF and TF-FVIIa were also analyzed. It was seen that on superimposing each of the three toxins docked with TF, ringhalexin and najalexin resulted in an RMSD of 1.035 Å, ringhalexin and ophiolexin resulted in an RMSD of 0.818 Å, and najalexin and ophiolexin resulted in an RMSD of 0.914 Å. On superimposing each of the three toxins docked with TF-FVIIa, ringhalexin and najalexin resulted in an RMSD of 0.575 Å, ringhalexin and ophiolexin resulted in an RMSD of 1.450 Å, and najalexin and ophiolexin resulted in an RMSD of 1.786 Å. The changes in orientation among the three toxins on docking with TF and TF-FVIIa have significant implications on their interactions and binding free energies (discussed below).

#### Overview of Interaction of Toxins with TF and TF-FVIIa

As expected, all three closely related toxins bind to the same site on TF and TF-FVIIa. In both cases of TF and TF-FVIIa, the toxins interact with the extracellular NH _2_ -terminal segment of TF (1–219 residues) which is composed of two fibronectin type III domains. This part of TF also takes part in the complex formation with FVIIa and increases its activity toward natural substrates FIX, FX, and FVII. 50 The crystal structure of TF-FVIIa 51 shows that TF binding leads to conformational changes in the 170-loop (residues 170–178) in the heavy chain of FVIIa (serine-protease domain) resulting in its enhanced ability to activate FX. 50 52 53 Thus, the 170-loop plays a crucial role in the formation of TF-FVIIa complex. The docking results revealed that all toxins use loops II and III, while ophiolexin also uses loop I to interact with TF alone. However, their interactions with TF shift to loops I and II in TF-FVIIa complex and the loop III interactions with heavy chain of FVIIa. It was interesting to note that all three toxins showed interactions with residue Gln170 of the 170-loop of FVIIa (for details, see below). Thus, the interactions so close to 170-loop may explain the inhibition of FX activation by these orphan group I 3FTxs.

#### Molecular Interactions with TF

**Fig. 6:**
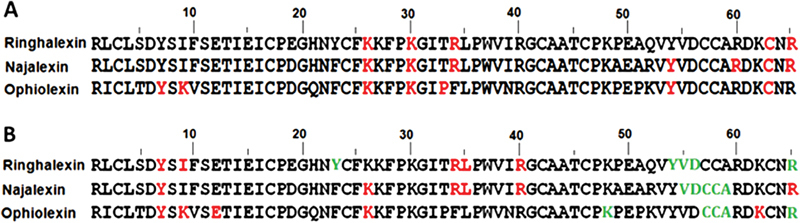
Amino acid residues of ringhalexin, najalexin, and ophiolexin involved in interactions with the extrinsic tenase complex. The residues involved in interactions with ( **A** ) TF and ( **B** ) TF-FVIIa. The residues involved in interactions with TF and the heavy chain of FVIIa have been highlighted in red and green, respectively.

**Fig. 7:**
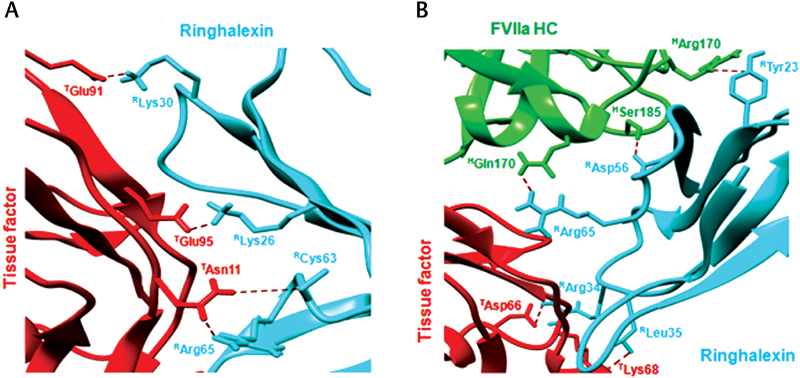
Contact surface and binding interactions of ringhalexin. Ringhalexin residues involved in interaction with ( **A** ) TF and ( **B** ) TF-FVIIa. All proteins are shown as ribbon structures with interacting residues as sticks. Color codes: cyan, ringhalexin; green, FVIIa HC; red, tissue factor.

Ringhalexin and najalexin have similar interaction interfaces on docking with TF ( Fig. 6 ). Docking results showed that three basic residues Lys26, Lys30, and Arg34 from loop II of both toxins form salt bridges, respectively, with Glu95, Glu91, and Thr40 in TF. However, najalexin appears to interact with TF using four residues Tyr54, Arg60, Cys63, and Arg65 (from loop III and the C-terminal) compared with only two residues Cys63 and Arg65 of ringhalexin ( Table 2 , Figs. 7A ; see also Supplementary Fig. S6A , C ). This difference could be due the presence of Pro49 in loop III of ringhalexin. Thus, substitution of Pro49 by Ala in ringhalexin appears to lead to stronger interaction. Lys9, present only in ophiolexin, is responsible for a salt bridge interaction with Glu99 of TF ( Table 2 ). This salt bridge leads to the interaction of Tyr7 with Thr13 of TF through an H-bond. The presence of hydrophobic Ile9 in ringhalexin and najalexin does not allow these interactions. In loop II of ophiolexin, Lys30 retains the interaction with Glu91 of TF, but presence of Pro33 alters the interaction of Lys26. Pro33 shows a salt bridge interaction with Arg74 of TF. Interestingly, the loop III of ophiolexin interacts with TF only through Tyr54 and Cys63. The alteration of interactions compared with ringhalexin could be due to the presence of Pro51. Thus, from the *in silico* studies, we speculate that the replacement of Pro49 and Pro51 in ophiolexin by Ala could further improve its interaction with TF with four residues from loop III. The percentage of buried residues in ringhalexin (47.53%) was less than that for najalexin (50.51%) and ophiolexin (51.56%) ( Table 2 ).

#### Molecular Interactions with TF-FVIIa

When toxins bind to TF-FVIIa, heavy chain of FVIIa interacts with loop III of respective toxins and provides a “molecular push” leading to slight variation of binding poses of toxins to TF. This push results in minor twist of 45 to 60° in the orientations of the three toxins docked with TF-FVIIa compared with TF only ( Fig. 5 ; see also Supplementary Fig. S5 ). The change in orientations and the presence of FVIIa leads to the differences that are observed in interactions of the three toxins with TF-FVIIa compared with the interactions with TF only. There is no sequence difference in loop I of ringhalexin and najalexin but still there is a difference in their ability to interact with TF; ringhalexin interacts with Tyr7 and Ile9, while najalexin interacts with only Tyr7. This could be due to changes in the orientation of these two toxins. Loop II in najalexin and ringhalexin has common H-bond interactions with TF ( Table 3 ). On docking the three toxins with TF-FVIIa, it was found that loop I of ophiolexin forms three H-bond interactions with TF, compared with two in ringhalexin and one in najalexin ( Table 3 , Fig. 7B ; see also Supplementary Fig. S6B , D ). The loop I of ophiolexin interacts with TF with Tyr7, Lys9, and Glu12. The presence of Lys9 along with Val10 allows Glu12 to interact with Asn11 of TF. In contrast, the replacement of Lys9 and Val10 in ringhalexin and najalexin appears to lead to poor interaction of their loop I with TF in TF-FVIIa complex. The presence of Pro33 and Phe34 in loop II of ophiolexin limits its interaction with TF through only Lys 26 ( Fig. 6 ). Ringhalexin and najalexin, on the other hand, interact with TF with Arg34, Leu35, and Arg40 residues. Najalexin also interacts with TF through Lys26 ( Fig. 6 ). Although the loop I and loop II residues of the three toxins are mostly responsible for interacting with TF in TF-FVIIa complex, residues in loop III such as Arg65 in najalexin and Lys62 in ophiolexin also interacts with TF. This could be because of the differences in main chain orientation caused by the absence of Pro49 in najalexin and presence of Pro51 in ophiolexin.

Loop III is mostly responsible for the interactions with FVIIa heavy chain in TF-FVIIa ( Fig. 6 ). Only ringhalexin uses Tyr23 in loop II to interact with Arg170 of FVIIa heavy chain ( Table 3 ). Although there is not much difference in the sequence of loop III in najalexin and ringhalexin, they interact with FVIIa heavy chain with different residues. These differences in their interactions with FVIIa heavy chain are probably due to the presence of Pro49 in ringhalexin ( Fig. 6 ). The presence of Pro47, Pro49, and Pro51 in ophiolexin allows five residues Lys48, Cys57, Cys58, Ala59, and Arg65 of the loop III to interact with the FVIIa heavy chain. As expected, the percentage of buried residues in ringhalexin (49.29%) was less than that for najalexin (51.78%) and ophiolexin (53.18%) ( Table 3 ).

#### Differences in Interactions Affect the Binding Free Energy

The Δ binding free energy was the lowest for TF-FVIIa, indicating that toxins form the most stable complex with TF-FVIIa. On docking the three toxins with TF, the additional interactions through loop I residues of ophiolexin appear to be responsible for the lowest Δ total binding free energy among them (−94.731 kcal/mol, compared with −82.188 kcal/mol for najalexin and −76.612 kcal/mol for ringhalexin). Overall, ophiolexin and najalexin exhibit more interactions in loop I and loop III, respectively, compared with ringhalexin. Therefore, Δ total binding free energy resulting from docking ophiolexin with TF-FVIIa (−379.706 kcal/mol) and najalexin (−305.777 kcal/mol) was lower than that of ringhalexin (−211.326 kcal/mol).

#### Functional Residues Involved in Binding to TF-FVIIa

*In silico* studies revealed that orphan group I 3FTxs interact with TF-FVIIa through functional residues in all three loops. In loop I, Tyr7, Lys9, and Glu12 play important role in binding to TF, while the replacement of Phe10 by smaller hydrophobic Val10 may be essential. In loop II, Lys 26, Arg33, Leu34, and Arg40 may play crucial role in the interaction with TF. The presence of Pro33 and Phe34 appears to hinder these interactions. Val 55, Asp56, Cys57, Cys58, Ala59, and Arg65 from loop III and C-terminal play critical role in interactions with FVIIa heavy chain. The presence of Pro49 and Pro51 appear to affect these interactions. These functional site residues are not found in other functional classes of 3FTxs.

### Alanine Mutagenesis Reveals the Important Residues

#### Effects of Mutation of Ringhalexin Residues

**Fig. 8:**
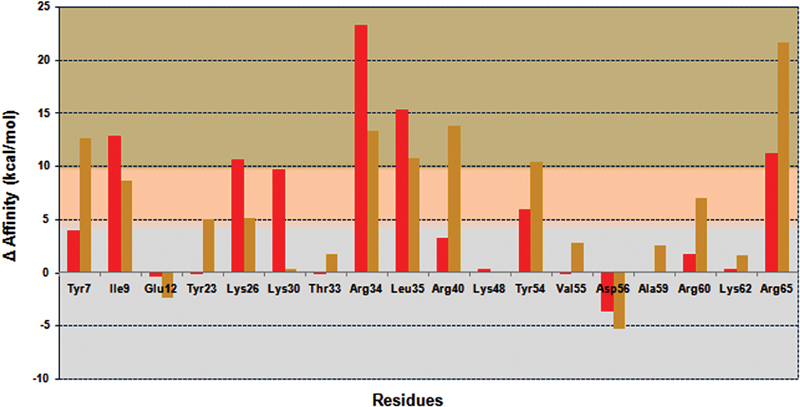
Alanine scan and comparison of Δ binding affinity. The plot shows the comparison of Δ binding affinity on mutation of residues involved in interactions of ringhalexin–TF and ringhalexin–TF-FVIIa. Color codes of bars: red, TF; gold, TF-FVIIa. Gradations: no significance < 5 kcal/mol; significant > 5 kcal/mol; strong >10 kcal/mol.

To analyze the effects of mutation on the binding affinity of ringhalexin toward TF, all functional site residues involved in interactions were mutated. Ala (or Gly for original Ala residues) scanning of the residues of ringhalexin was performed using *in silico* alanine scanning module and the difference in Δ binding affinity between native and mutated toxins in respective complexes was obtained. For mutation analysis, we assumed that there was no significant effect in binding affinity if the difference was <5 kcal/mol, a significant effect if >5 kcal/mol, and a strong effect if >10 kcal/mol. In the ringhalexin–TF complex, residues Tyr7, Arg40, Lys48, Val55, Arg60, Lys62, and Ala59 did not show any significant change in the Δ binding affinity after being mutated ( Fig. 8 ), as these residues were not involved in any interactions ( Table 2 ). Residues Ile9, Lys26, Arg34, Leu35, and Arg65 showed strong effects after mutation. These data correlate with the docking results as most of these residues were involved in either H-bond or salt bridge interactions of ringhalexin with TF. In the case of TF-FVIIa, ringhalexin residues Lys30, Thr33, Lys48, Lys62, and Ala59 did not show any significant changes in the Δ binding affinity after being mutated ( Fig. 8 ). These residues in ringhalexin were not responsible for any interactions with TF-FVIIa complex ( Table 3 ). Residues Ile9 and Tyr23 forming H-bond interactions with TF and FVIIa heavy chain residues, respectively, showed significant effect after mutation. Residues Tyr7, Arg34, Leu35, Arg40, Tyr54, and Arg65 involved in H-bond interactions with TF-FVIIa after mutation showed strong effects. However, residues Glu12 and Asp56 on mutation to Ala improved the binding affinity of ringhalexin toward both TF and TF-FVIIa.

#### Effects of Mutation of Najalexin Residues

In najalexin, mutation of residues Tyr7, Phe23, Thr33, Leu35, Arg40, Lys48, Val55, Ala59, and Lys62 did not have any significant effects on the Δ binding affinity of the najalexin–TF complex ( Supplementary Fig. S7A ). This makes sense, as these residues were not involved in any interactions ( Table 2 ). Residues Lys26, Arg34, Tyr54, Arg60, and Arg65, which form either salt bridge or H-bond interactions, show strong effects after mutation. In the case of TF-FVIIa, residues Val55 and Ala59 (which form H-bonds with FVIIa heavy chain in TF-FVIIa) also showed negligible effects after mutation ( Table 3 ). Mutations of najalexin residues Tyr7, Ile9, Phe23, Lys26, and Arg60 showed significant effects, among which Tyr7 and Lys26 were involved in H-bond interactions with TF in TF-FVIIa ( Table 3 ). Furthermore, mutation of residues Arg34, Leu35, Arg40, and Arg65 (all involved in H-bond interactions with TF in TF-FVIIa) showed strong effects on binding affinity. Similar to ringhalexin, residues Glu12 and Asp56 of najalexin on mutation to Ala improved the binding affinities.

#### Effects of Mutation of Ophiolexin Residues

In the case of ophiolexin, mutation of residues Phe23, Phe34, Leu35, Arg40, Lys48, Val55, Ala59, Arg60, Lys62, and Arg65 did not show any significant changes in the Δ binding affinity in the ophiolexin-TF complex ( Supplementary Fig. S7B ). These residues also were not involved in any interactions ( Table 2 ). Ophiolexin residues Tyr7, Lys9, Lys26, Lys30, and Tyr54, involved in either salt bridge or H-bond interactions, resulted in strong effects after mutation. Although ophiolexin residues Lys48 and Ala59 showed H-bond interactions with FVIIa heavy chain in TF-FVIIA complex, they had negligible effects after mutation. Mutation of residues Tyr7, Lys9, Glu12, Lys26, and Lys62 (which form H-bond interactions with TF in TF-FVIIa) showed significant effects on binding affinity. Residue Arg65 in loop III of ophiolexin, which forms H-bond interactions with FVIIa heavy chain, showed a strong effect after mutation. Similar to ringhalexin, residue Asp56 of ophiolexin on mutation improved binding affinities.

Using molecular docking and mutation studies, we have identified the *in silico* functional site of orphan group I 3FTxs. We speculate that substitution of residues Pro49 and Pro51 by Ala might lead to better interactions with TF. These studies may help in designing potent inhibitors of the extrinsic tenase complex. Further studies are needed to validate these findings through recombinant expression of several mutants and the evaluation of their inhibitory properties and binding to TF and TF-FVIIa complex are underway.

### Conclusion

Our *in silico* studies suggest that all three toxins of orphan group I showed the highest binding affinity (lowest binding free energy) toward TF-FVIIa complex by forming H-bond and salt bridge interactions with residues of the TF and FVIIa, respectively. The residues, crucial for the binding interactions of these toxins with TF-FVIIa, have been identified asTyr7, Lys9, Glu12, Lys26, Arg34, Leu35, Arg40, Val55, Asp56, Cys57, Cys58, and Arg65. These studies help in understanding the structure–function relationships of this group of toxins and their anticoagulant role of inhibiting FX activation by the extrinsic tenase complex.
