# Supplementary material for: Orphan Three-Finger Toxins Bind at Tissue Factor–Factor VIIa Interface to Inhibit Factor X Activation: Identification of Functional Site by Docking
Source: TH Open. 2018 Sep 26;2(3):e303–14. doi: 10.1055/s-0038-1672184 (PMC6524886; doi:10.1055/s-0038-1672184)
Supplement: Supplementary file 1 — Supplementary Material [file 10-1055-s-0038-1672184-s180038.pdf]

Supplementary Figures

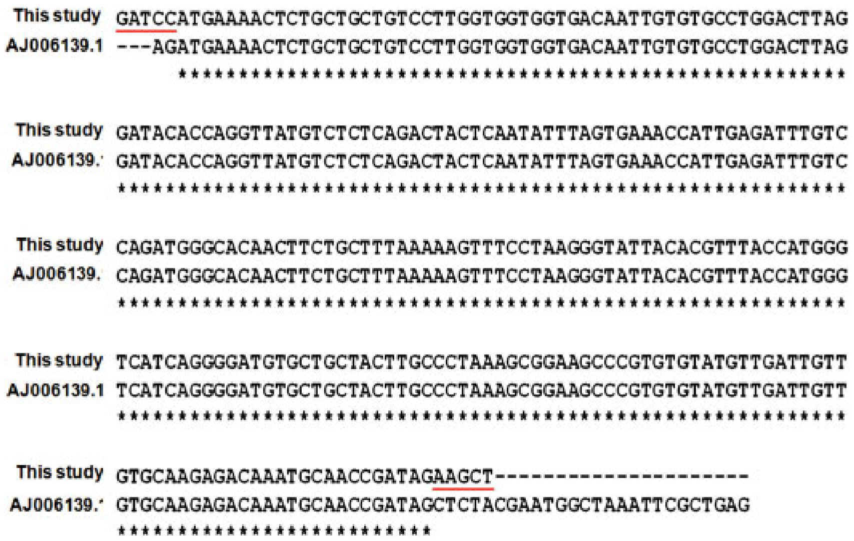

Supplementary Fig. S1 Complete sequence of najalexin clone. The sequence is aligned to natralexin sequence from *Naja atra* (AJ006139.1).

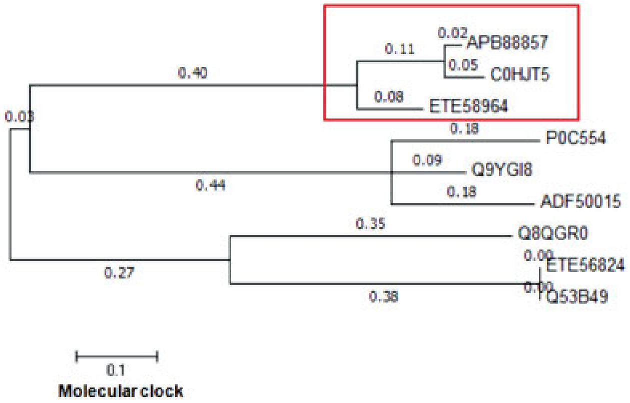

Supplementary Fig. S2 Phylogenetic analysis of the 3FTxs. The tree shows evolutionary distances between najalexin (APB88857) and its homologs. The numbers indicate the time since deviation for the respective node. The proteins are indicated by NCBI accession numbers.

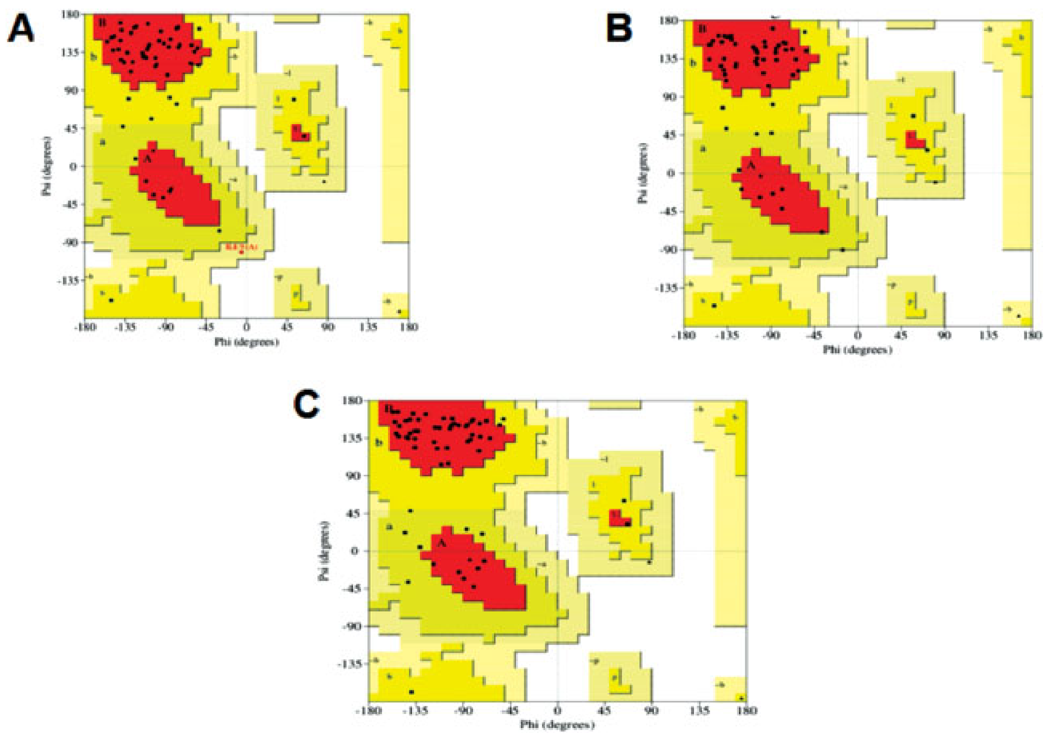

Supplementary Fig. S3 Evaluation of molecular models of orphan group I 3FTxs using Ramachandran plot. Phi–Psi stereochemical profiles of (**A**) ringhalexin, (**B**) najalexin, and (**C**) ophiolexin. All the residues were in the allowed regions, with 80, 89.3, and 86.8% of the amino acid residues falling in the most favored regions, respectively.

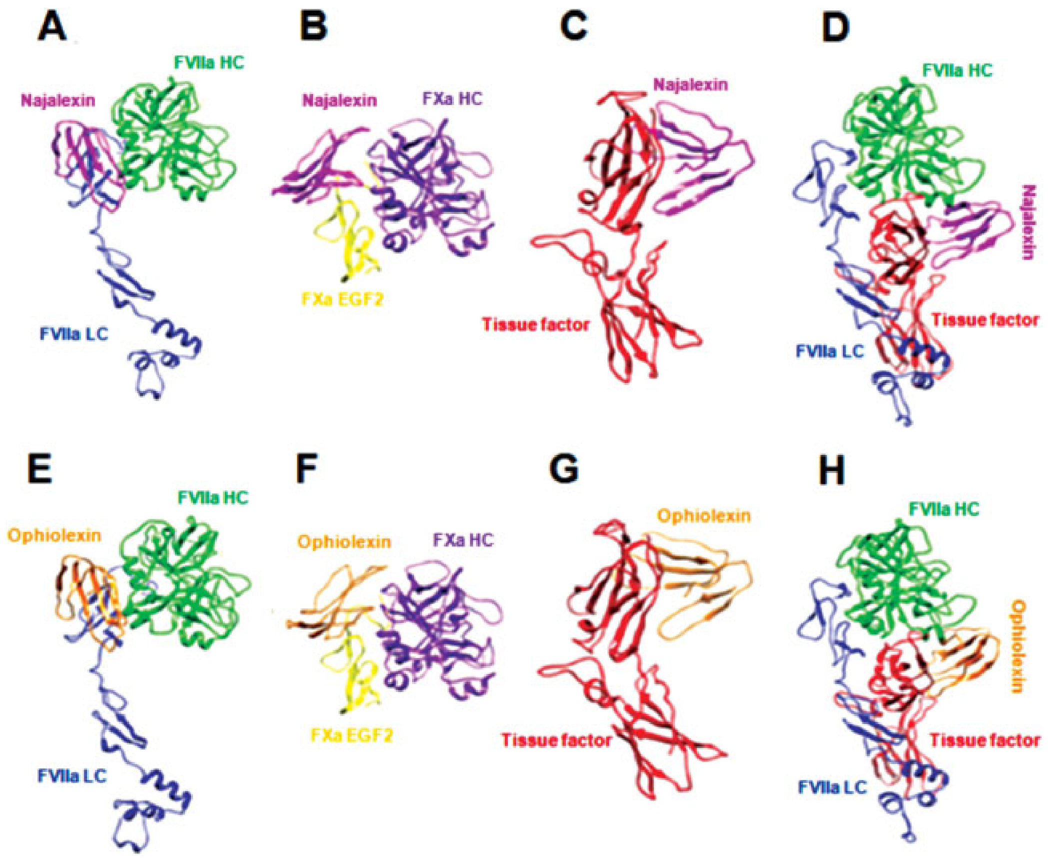

Supplementary Fig. S4 Molecular docking of najalexin and ophiolexin with coagulation factors of the extrinsic tenase complex. Interactions of najalexin with (**A**) FVIIa, (**B**) FX, (**C**) TF, and (**D**) TF-FVIIa, and ophiolexin with (**E**) FVIIa, (**F**) FX, (**G**) TF, and (**H**) TF-FVIIa. HC, heavy chain; LC, light chain.

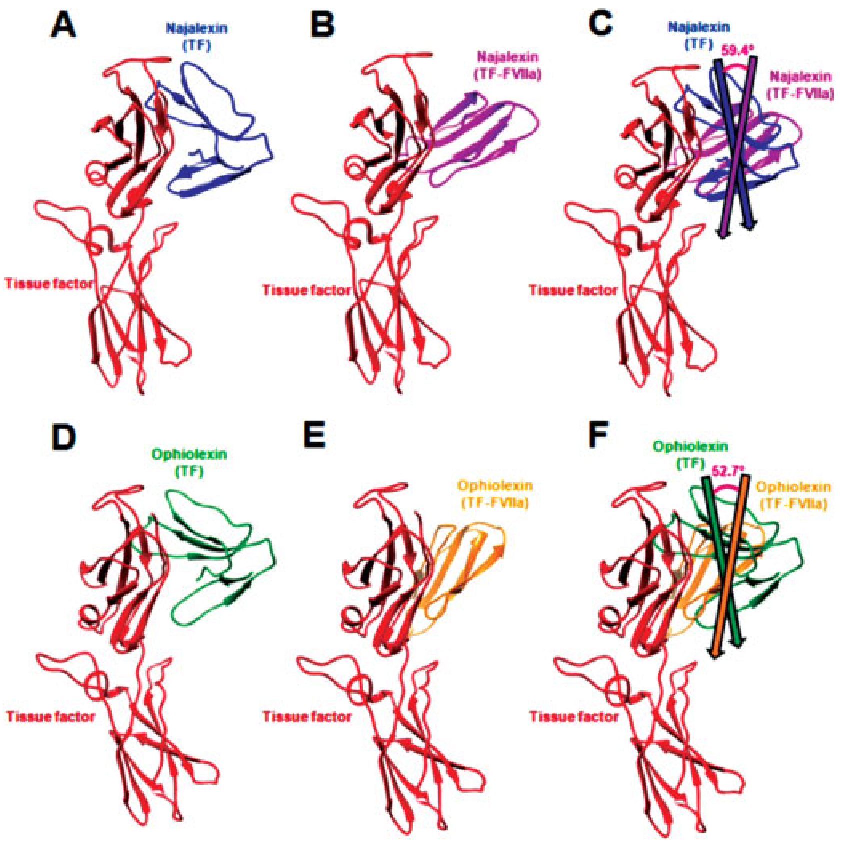

Supplementary Fig. S5 Change of orientation between najalexin and ophiolexin docked with TF and TF-FVIIa. Binding poses of najalexin and ophiolexin to (**A**, **D**) TF and (**B**, **E**) TF-FVIIa, respectively. (**C**, **F**) Superimposition of binding poses of ringhalexin and ophiolexin docked with TF and TF-FVIIa results in an RMSD 0.714 and 0.742 Å. The vectors passing through bound ringhalexin and ophiolexin to TF and TF-FVIIa make an angle of 59.4 and 52.7 degrees.

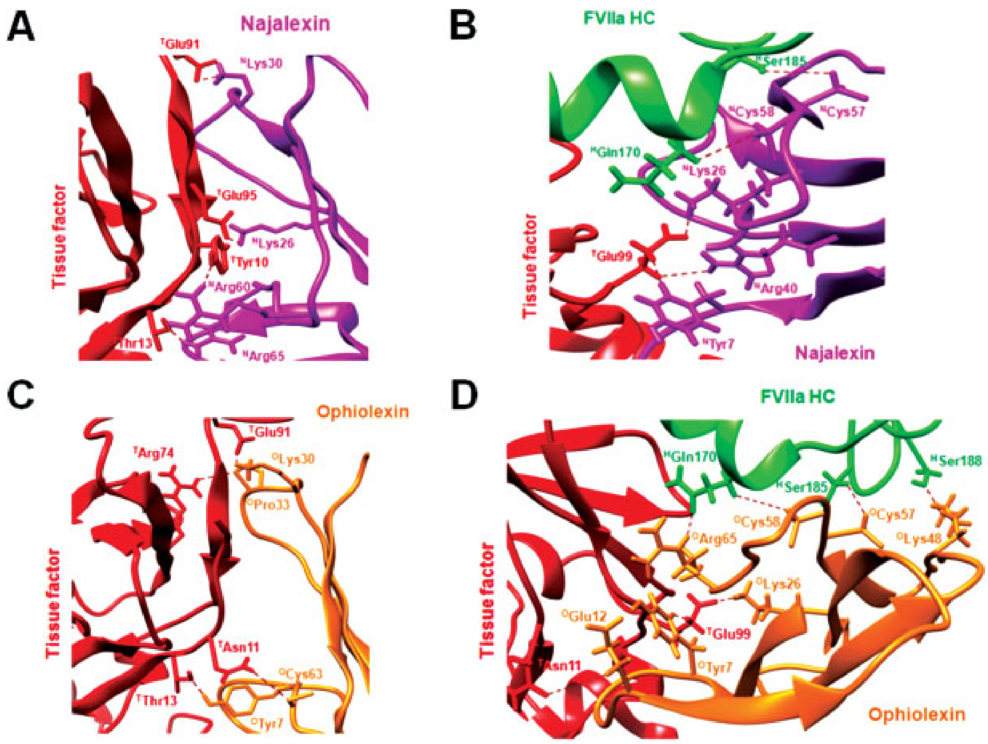

Supplementary Fig. S6 Contact surface and binding interactions of ringhalexin and ophiolexin. Ringhalexin and ophiolexin residues involved in interaction with (**A**, **C**) TF and (**B**, **D**) TF-FVIIa, respectively. All proteins are shown as ribbon structures with interacting residues as sticks. Color codes: magenta, najalexin; orange, ophiolexin; green, FVIIa HC; red, tissue factor.

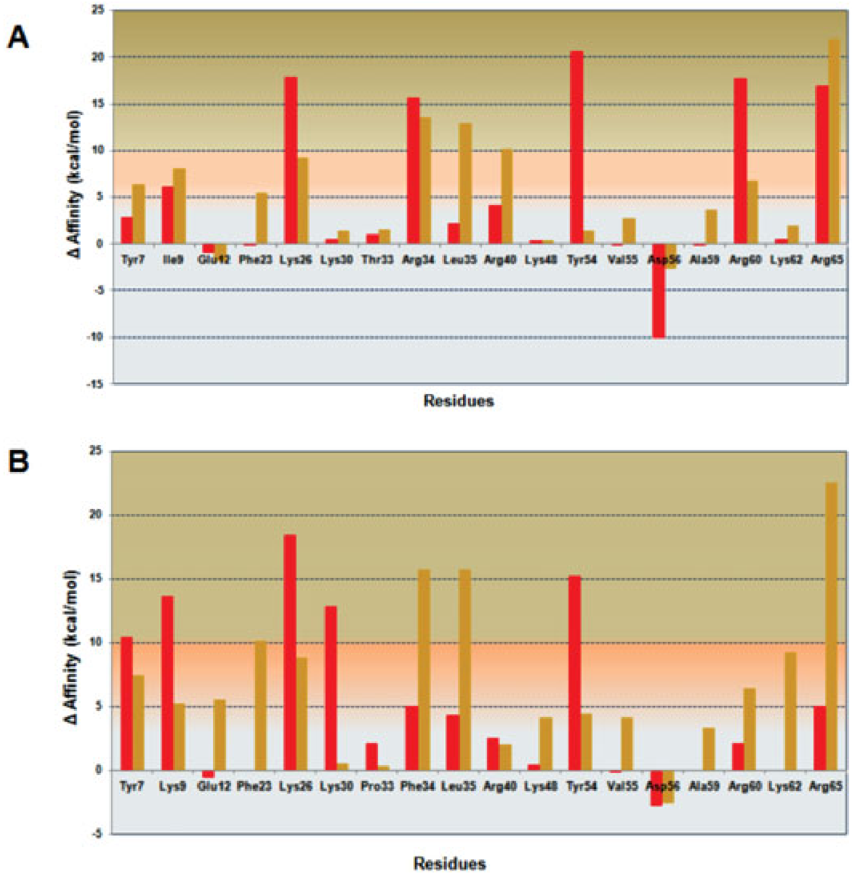

Supplementary Fig. S7 Alanine scan and comparison of Δ binding affinity. The plot shows the comparison of Δ binding affinity on mutation of residues involved in interactions of (**A**) ringhalexin–TF and ringhalexin–TF-FVIIa and (**B**) najalexin–TF and najalexin–TF-FVIIa, respectively. Color codes of bars: red, TF; gold, TF-FVIIa. Gradations: no significance $<$ 5 kcal/mol; significant $>$ 5 kcal/mol; strong $>$10 kcal/mol.
